# Preventive efficiency of Cornelian cherry (*Cornus mas* L.) fruit extract in diniconazole fungicide-treated *Allium cepa* L. roots

**DOI:** 10.1038/s41598-021-82132-4

**Published:** 2021-01-28

**Authors:** Tuğçe Kalefetoğlu Macar, Oksal Macar, Emine Yalçιn, Kültiğin Çavuşoğlu

**Affiliations:** 1 Department of Food Technology, Şebinkarahisar School of Applied Sciences, Giresun University, 28400 Giresun, Turkey; 2 Department of Biology, Faculty of Science and Art, Giresun University, 28049 Giresun, Turkey

**Keywords:** Environmental impact, Genetics, Risk factors

## Abstract

Cornelian cherry (*Cornus mas* L.) is a medicinal plant with antioxidant-rich fruits. Diniconazole, a broad-spectrum fungicide, is employed extensively. The present study was designed to evaluate the preventive efficiency of *C. mas* fruit extract (CME) against the toxic effects of diniconazole on a model organism, *Allium cepa* L. For this aim, physiological, cytogenetic and biochemical parameters as well as the meristematic cell damages were investigated in *A. cepa* treated with diniconazole and *C. mas* extract. *A. cepa* bulbs were divided into six groups which were treated with tap water, 0.5 g/L CME, 1.0 g/L CME, 100 mg/L diniconazole, 0.5 g/L CME + 100 mg/L diniconazole and 1.0 g/L CME + 100 mg/L diniconazole, respectively. Diniconazole application caused a significant reduction in germination percentage, root elongation and total weight gain. Mitotic index decreased, while chromosomal aberrations increased following diniconazole application. Diniconazole caused significant rises in malondialdehyde content and the total activities of superoxide dismutase and catalase enzymes. The meristematic cell damages induced by diniconazole were indistinct transmission tissue, epidermis cell deformation, thickening of the cortex cell wall and flattened cell nucleus. Aqueous *C. mas* extracts induced a dose-dependent prevention and amelioration in all damages arisen from diniconazole application.

## Introduction

Agriculture is a vital spot for economic development for many countries. Since plant diseases, weeds and pests are the most considerable concerns for adequate crop production worldwide, the pest control is a crucial step for eliminating the yield losses^1^. Pesticides are biocidal substances which have been indispensably utilized in crop production for an effective fight against pests such as insects, fungi, herbs and mites^2^. Despite their large scale uses and economic benefits in agricultural areas, overexposure to various pesticides carries high risks for the environment and biota.

As a multi-member pesticide family, triazoles have become one of the most commonly applied fungicides due to their effectiveness, systemic motion, wide spectrum, and versatile usage for suppressing plant diseases and yield loss due to pathogenic fungi^3,4^. Scientific efforts have focused on unwanted health risks on untargeted organisms caused by repeated and global employment of fungicides^5^.

Diniconazole [(*E*)‐(*RS*)‐1‐(2,4,‐dichlorophenyl)‐4,4‐dimethyl‐2‐(1*H*‐1,2,4‐triazole‐1‐yl)pent‐1‐en‐3‐ol] is a triazole type with an excellent antifungal capacity in protection of fruits, vegetables, cereals and tea plants against several diseases through therapeutic and preventive practices^6^. Plant diseases such as brown rust, powdery mildew, *Septoria* and scab formation are only a few of the problems in which the diniconazole fungicide is employed^7^. It is classified as a systemic fungicide that functions in the inhibition of the ergosterol synthesis, particularly in 14α-demethylation step of the biosynthesis via lanosterol-14 α-ademethylase enzyme^6^. Xu et al.^4^ stated that its long half-life duration and highly lipophilic character let diniconazole fungicide have crucial risk potential for untargeted organisms. Although it is widely used in agricultural production processes, diniconazole was proven to be a suppressor for plant growth, chlorophyll synthesis and photosynthesis^8–11^. According to the studies on experimental animals, exposure to diniconazole is resulted in thyroid, liver dysfunctions, kidney failures, tachycardia and malformed fetus formation^12,13^. Besides, it causes a serious induction in reactive oxygen species (ROS) generation and apoptosis in human cells^4^.

Cornelian cherry (*Cornus mas* L.) is a plant with olive-shaped, scarlet-coloured and sour-tasting fruits that has been cultivated in gardens for 4000 years. It is recently grown in Asia and the interior of the European continent^14^. Cornelian cherry fruits are famous for being endowed with antibacterial, anticancer, antioxidant, antidiabetic, anti-inflammatory, and hypoglycemic functions^15^. Additionally, the fruits are frequently used in the medical and traditional treatments of diarrhea and other gastrointestinal disorders in Turkey^16^. As valuable sources of anthocyanins, flavonoids, phenolic acids, iridoids, terpenoids and Vitamin C, Cornelian cherry fruits possess marvelous nutritional and health-promoting virtues^17,18^. In Turkey, 10,269 tons of Cornelian cherry fruits were harvested from 686,000 trees in 2019^19^. However, the number of these trees is gradually decreasing due to the fact that the fruits cannot be used commercially enough^18^.

Scientists from all over the world use various model organisms in order to test chemicals which are potentially toxic to untargeted organisms. *Allium cepa * L., a test material approved by the Environmental Protection Agency, the United Nations Environmental Programme and the World Health Organization, is a simple model used to assess the cytotoxicity and genotoxicity of risky pollutants^20^. It has been utilized since the 1920s due to its cells with large and well stained chromosomes (2n = 16)^21^. Indeed, *A. cepa* assay provides adequate results in studies following major endpoints in genetic damages including chromosomal aberrations (CAS) and micronuclei (MN)^22^. Owing to its fast growing roots, *A. cepa* bulbs provide satisfying data on the relationship between genotoxicity and oxidative stress along with the growth-retardation effect of a pollutant.

Studies dialed with the toxicity of diniconazole fungicide on untargeted organisms are extremely scarce^12,13^. In addition, there is a lack of studies in the literature on the potential use of Cornelian cherry against the harmful effects of environmental pollutants. Therefore, the objective of this study was to investigate the cytotoxic and genotoxic effects of diniconazole application besides the preventive efficiency of Cornelian cherry in diniconazole-treated *A. cepa* roots. For this purpose, some physiological, cytogenetic and biochemical parameters including germination percentage, root elongation, weight gain, CAs incidence, MN formation, mitotic index (MI) fluctuation, lipid peroxidation and alterations in antioxidant enzyme activities were analyzed. Furthermore, meristematic destructions caused by the application of diniconazole were observed.

## Materials and methods

### Experimental arrangement of materials

*A. cepa* bulbs purchased from local markets were selected to be approximately equal in weight (4.10–4.32 g). Dry bases on the stem were scraped immediately after the scale leaves were removed in order to reach the primordial roots. Bulbs were separated into six groups (n = 50) as seen in Table 1.

**Table 1:** Experimental layout of the groups.

Groups	Treatments
Group 1 (control)	Tap water
Group 2	0.5 g/L CME
Group 3	1.0 g/L CME
Group 4	100 mg/L diniconazole
Group 5	0.5 g/L CME + 100 mg/L diniconazole
Group 6	1.0 g/L CME + 100 mg/L diniconazole

Ripe Cornelian cherry fruits were collected manually in September 2019 in the Şebinkarahisar District of Giresun in Turkey. The fruits were brought to the laboratory and washed thoroughly with water to remove the residual dirt and dust. Fruit seeds were removed before the aqueous extract was prepared. Fruits were air dried at room temperature for 1 week in a dark chamber and stored at − 18 °C until the experiment started. Approximately 87 g of dry fruit was obtained from 400 g of fresh fruit without seeds. Fully dried fruits were powdered with a grinder and homogenized in distilled water with ultrasonic bath for 1 h at room temperature. The homogenate filtered through paper filter (Whatman filter No. 1) and freshly prepared *C. mas* extract (CME) was used for treatments.

Diniconazole solutions were prepared using 1% diniconazole with trade name FERNEX DS purchased from Fertil Chemistry, Konya, Turkey. Application dose of diniconazole solution (100 mg/L) was determined considering the previous study of Demirtaş et al.^23^.

Treatments were carried out with six groups consisting of 50 bulbs each. Glass tubes in which bulbs were placed were kept for 72 h at 24 ± 1 °C in a dark room throughout the experiment. The control group (Group 1) was dipped in tap water during the test period, while Group 2, Group 3, Group 4, Group 5 and Group 6 were treated with 0.5 g/L CME, 1.0 g/L CME, 100 mg/L diniconazole, 0.5 g/L CME + 100 mg/L diniconazole and 1.0 g/L CME + 100 mg/L diniconazole, respectively.

### Physiological analyses

The total weight gains (g) of *A. cepa* bulbs was calculated by subtracting their initial weights from the last weights measured at the end of the experiment period. 10 bulbs were used to determine the average weight increase of each group.

Germination percentages of the groups (%) were determined by screening the roots sprout up from the stems (Eq. 1)^24^. In this study, emergence of the roots was considered as germination of the bulbs and 50 bulbs were used to determine the germination percentage of each group.

1 $$ {\text{Germination}}\;{\text{ percentage}}\;\left( \% \right) = \left[ {{\text{Number}}\;{\text{of}}\;{\text{germinated}}\;{\text{members}}\;{\text{of}}\;{\text{each}}\;{\text{group}}/{\text{Number}}\;{\text{of}}\;{\text{total}}\;{\text{members}}\;{\text{of}}\;{\text{each}}\;{\text{group}}} \right] \times 100.$$

Average root growth of the groups was determined with a ruler at the end of the treatment period. While evaluating the root elongation level of each group, a total of 100 adventitious roots randomly selected from 10 different bulbs were taken into consideration.

### Genotoxicity assay

Genotoxic effects of diniconazole fungicide on *A. cepa* roots were evaluated considering the MI, MN and CAs. For genotoxicity analyses, root cells were prepared according to the assay mentioned by Staykova et al.^25^. 1 cm long pieces were cut from the root tips and fixed with glacial acetic acid:ethanol (3:1) solution. The root tips were washed with ethanol (96%) after 2 h of fixation. 70% ethanol was used to store the fixed roots in a refrigerator at + 4 °C. The hydrolysis procedure was completed by keeping the samples in a 1 N HCl solution at 60 °C immediately after removing the root materials from the refrigerator. 2 mm long root tips were stained with 1% acetocarmine for 24 h in a clean flask and crushed in 45% acetic acid to prepare microscope slides. Genotoxicity tests were performed on 10 slides screened with a research microscope at 1000 × magnification for each group. 1000 cells from 10 slides were examined to determine CAs and MN in each group and MN was evaluated according to the rules specified by Fenech et al.^26^. The dividing cells in totally 10,000 cells from 10 slides were considered as the average MI level for each group.

### Biochemical analyses

Each biochemical analysis was repeated 3 times for each group:

#### Determination of SOD and CAT activities

SOD [EC 1.15.1.1] and CAT [EC 1.11.1.6] enzymes were extracted from 0.5 g of root material according to the method proposed by Zou et al.^27^. Roots cut and washed with distilled water were ground with liquid nitrogen using mortar and pestle until powdered. The homogenization of the root materials were achieved using 5 mL sodium phosphate buffer (50 mM, pH 7.8). Newly prepared homogenates were centrifuged at 14,000 rpm for 20 min at 4 °C to obtain supernatants containing the enzymes.

The method of Beauchamp and Fridovich^28^ was chosen to evaluate the total SOD activity of the groups. 0.01 mL enzyme extract was mixed with 1.5 mL sodium phosphate (0.05 M) buffer with pH 7.8, 0.3 mL methionine (130 mM), 0.3 ml riboflavin (20 μM), 0.3 ml EDTA-Na_2_ (0.1 mM), 0.3 mL nitroblue tetrazolium chloride (750 μM), 0.01 mL polyvinylpyrrolidone (4%) and 0.28 mL distilled water. The mixture was placed across the fluorescent light (375 µmol m^−2^ s^−1^) to initiate the catalysis. The absorbance of the samples was recorded at a wavelength of 560 nm after 15 min of illumination. The total SOD activity of each group calculated as unit per mg fresh weight^27^.

The method of Beers and Sizer^29^ was chosen to evaluate the total CAT activity of the groups. 0.2 ml enzyme extract was added to the reaction medium containing 1.5 ml sodium phosphate buffer (0.2 M, pH 7.8), 1.0 mL distilled water and 0.3 mL hydrogen peroxide (0.1 M). 0.2 ml enzyme extract was added to the mixture to initiate the enzymatic catalysis. The total CAT activity was recorded considering the decline in the absorbance of the sample at a wavelength of 240 nm. Enzymatic consumption of hydrogen peroxide was expressed as OD240 nm minute per gr fresh weight^27^.

#### Determination of malondialdehyde level

The procedure of Unyayar et al.^30^ was chosen to identify the lipid peroxidation expressed as malondialdehyde (MDA) level. 5% trichloroacetic acid used to homogenize 0.5 g of chipped root sample. The homogenization process was completed at room temperature using mortar and pestle until the roots became powdered. After centrifugation at 12,000 rpm for 15 min at room temperature, the same volume of supernatant added to a mixture of 20% trichloroacetic acid and 0.5% thiobarbituric acid. Freshly prepared mixture was heated at 95 °C for half an hour. The reaction occurred at high temperature was terminated by transferring the mixture to the ice bath. After centrifugation at 10,000 rpm for 5 min, the absorbance of the supernatant was recorded at 532 nm. The average MDA amounts of the groups were calculated utilizing the specific extinction coefficient (155 M^−1^ cm^−1^) and presented as μM per g fresh weight.

### Determination of meristematic damages

Cross sections of the roots treated with water and diniconazole solution were used to investigate meristematic damages of the fungicide. After the root tips were washed thoroughly with distilled water for 3 min, the root caps were removed. Decapitated roots were cross-sectioned manually using a razor. 5% methylene blue was dropped onto the root slices in order to stain the cells. Microscope slides were examined under × 500 zoom for meristematic disintegration.

### Analysis of data

Results were presented as mean ± standard deviation. The data were subjected to one-way ANOVA and Duncan’s test to assess the statistical significance (p < 0.05) between the means.

## Results and discussion

The physiological effects of diniconazole fungicide and CME on *A. cepa* bulbs were examined with respect to germination, root growth and weight increase parameters (Table 2). There was no remarkable difference between the groups treated with CME (Groups 2 and 3) and the control group treated with tap water in all three parameters. Group 4 exhibited a significant growth delay due to diniconazole administration when compared to the first three groups. Indeed, the germination percentage in diniconazole-treated Group 4 almost halved, while the mean root length dropped to 1.60 ± 0.44 cm. The average weight gain in the control group was approximately 7.8 times that of the group treated with diniconazole. Our results were in accordance with Demirtaş et al.^23^ who reported that excessive dose of diniconazole fungicide induced a sharp reduce in germination, root elongation and total weight increase in *A. cepa* bulbs. Similarly, another study showed that diniconazole treatment led to a reduction of root and stem growth as well as germination ratio^8^. Yeoung et al.^31^ reported that stunted growth caused by triazoles including diniconazole occurs due to the inhibition of gibberellin-like phytohormone biosynthesis. On the other hand, the preventive effect of CME administered as a mixture with diniconazole against growth retardation elevated with increasing doses of the extract in Group 5 and Group 6. The germination percentage in Group 6 was approximately 1.5 times that of Group 4. Besides, the detrimental effects of diniconazole in root elongation and weight gain in Group 4 relieved more in Group 6 than that of Group 5. Studies on the preventive effects of CME against compounds those inhibit plant growth are not available in the literature. However, Gülçin et al.^32^ demonstrated that water extract of *C. mas* possessed extremely rich antioxidant capacity. In addition, Klymenko et al.^14^ emphasized that *C. mas* genotypes have a plenty of vitamins and phenolic compounds, providing various pharmacological and biological properties to the fruit. The positive effects of CME on diniconazole-related growth regression in *A. cepa* bulbs may be associated with its biologically active components.

**Table 2:** Physiological changes caused by diniconazole and CME.

Groups	Germination (%)	Root length (cm)	Initial weight (g)	Final weight (g)	Weight gain (g)
Group 1	98	6.50 ± 1.14^a^	4.10 ± 0.56	8.56 ± 1.74	+ 4.46^a^
Group 2	98	6.58 ± 1.15^a^	4.24 ± 0.58	8.62 ± 1.75	+ 4.38^a^
Group 3	99	6.64 ± 1.16^a^	4.32 ± 0.62	8.94 ± 1.78	+ 4.62^a^
Group 4	50	1.60 ± 0.44^d^	4.18 ± 0.57	4.75 ± 0.74	+ 0.57^d^
Group 5	61	2.40 ± 0.64^c^	4.30 ± 0.60	5.78 ± 0.96	+ 1.48^c^
Group 6	73	3.50 ± 0.88^b^	4.26 ± 0.56	6.80 ± 1.36	+ 2.54^b^

*Group 1: Control, Group 2: 0.5 g/L CME, Group 3: 1.0 g/L CME, Group 4: 100 mg/L diniconazole, Group 5: 0.5 g/L CME + 100 mg/L diniconazole, Group 6: 1.0 g/L CME + 100 mg/L diniconazole. The averages shown with different letters^(a–d)^ in the same column are statistically significant (p < 0.05).

**Figure 1:**
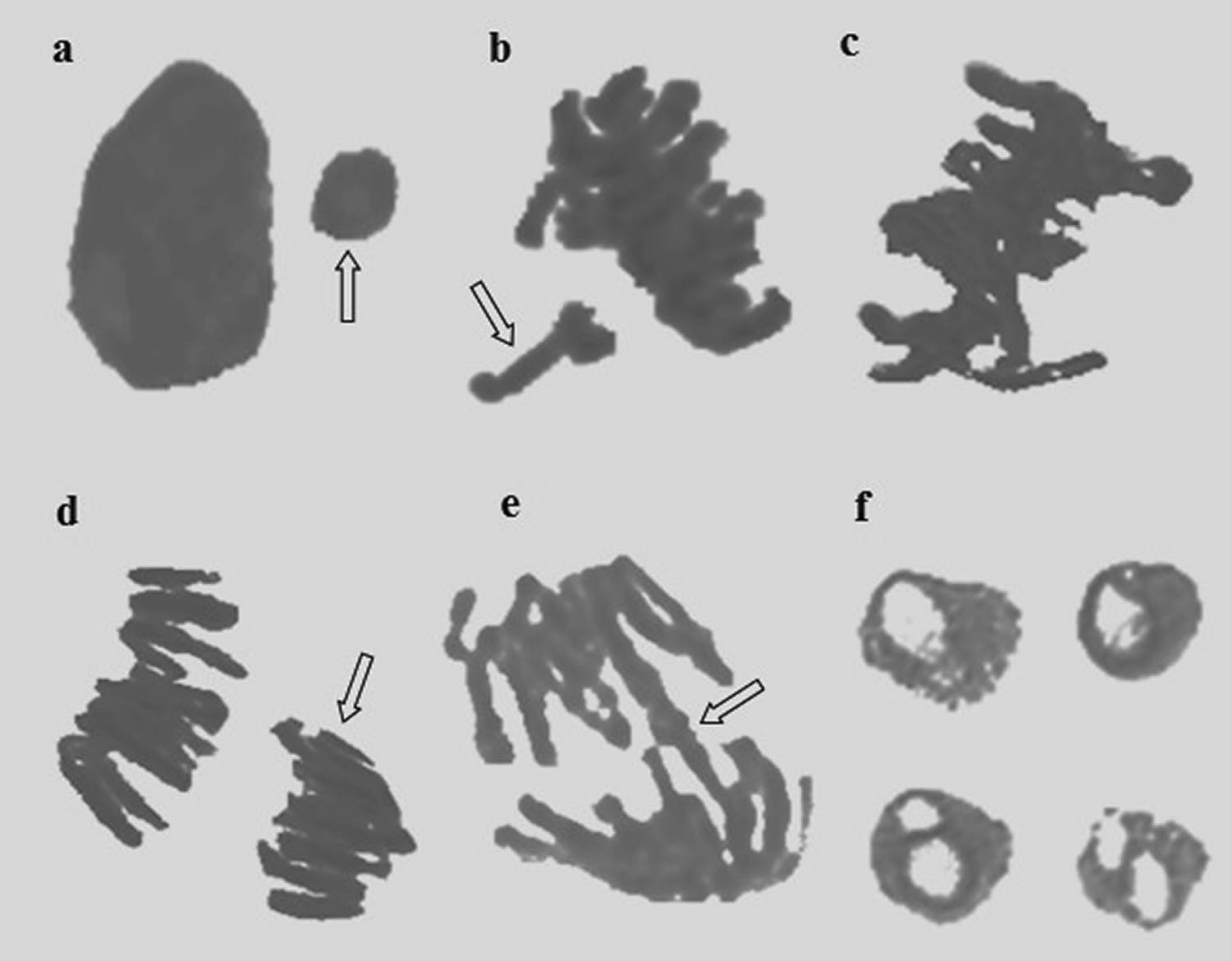
Chromosomal damage caused by diniconazole. (**a**) MN, (**b**) fragments, (**c**) sticky chromosome, (**d**) unequal distribution of chromatin, (**e**) bridge, (**f**) vacuole nucleus.

CME doses in Groups 2 and 3 did not induce any genotoxic effects on *A. cepa* bulbs when applied alone, similar to the control group (Table 3). On the other hand, a series of genotoxic malformations occurred in Group 4 following the diniconazole treatment. Genotoxic potential of diniconazole in cells was evidenced by a reduction in MI and increased amounts of CAs when compared to the control group. Chromosomal disorders occurred due to the administration of diniconazole were micronucleus, fragment, sticky chromosome, unequal distribution of chromatin and vacuole nucleus (Table 3, Fig. 1). MN frequencies of Group 1, Group 2 and Group 3 were 0.56 ± 0.64, 0.42 ± 0.54 and 0.24 ± 0.38, respectively. On the other hand, Group 4 had an extremely higher MN score than those of the first three groups (Fig. 1a). MN formation is a commonly preferred reflector in order to detect the clastogenic and aneugenic consequences of toxic compounds^33^. Fenech and Crott^34^ noted that failures in cell cycle could lead to the emergence of acentric fragments or disintegration of complete chromosomes to the main nucleus, which in turn resulted in MN formation. Here, we demonstrate that diniconazole treatment resulted in a terrific leap in fragment abundancy in Group 4 (Fig. 1b). Fragment was more intense than MN in diniconazole-treated roots. It can be considered that MN frequency can usually be found at lower amounts than the other CAs due to the fact that all of the fragments do not become a distinct micronucleus. Other CAs observed abundantly in Group 4 was sticky chromosomes (Fig. 1c). Stickiness, a chromatid type abnormality, is arisen from the inappropriate folding of inner chromatins into chromatids or depolymerization of chromosomes^21^. It is considered to be an irreversible chromosomal abnormality and usually result in death of the cells^35^. As it causes unequal separation of chromosomes into the daughter cells^36^, this deviation type is a sign of the aneugenic action of diniconazole. Unequal distribution of chromatin occurrence was induced following diniconazole treatment in Group 4 (Fig. 1d). Auti et al.^37^ reported that unequal distribution of chromatin occurs when cells encounter chemicals during the latter anaphase stage of the mitosis. In our study, bridge was other CAs recorded in large quantities as a result of fungicide treatment (Fig. 1e). Bridge formation and stickiness are closely related to each other since the previous one might be a consequence of sticky chromosome formation during the metaphase^38^. The least frequent CAs in diniconazole-treated group was vacuole nucleus (Fig. 1f). According to Sutan et al.^39^, this kind of anomaly is not only a reflector of a toxic compound that directly targets the cell nucleus, but also evidence of damage on DNA replication during S step of the cell division. Our results on CAs-formative potential of diniconazole in *A. cepa* roots cells were in agreement with those obtained from Demirtaş et al.^23^. They described the CAs types formed as a result of dicinonazole treatment in *Allium* roots as fragment, bridge, binucleus cell, C-mitosis, polarization abnormality, stickiness and unequally exchange of chromatins. So far, many researchers were focused on the chromosomal anomalies caused by other triazole compounds in the literature^40,41^ and our data showed great harmony with the results of their studies. MI is calculated to quantify the mitotic proliferation performance of the cells and estimate the cytotoxic potency of the compounds^42^. Khan et al.^43^ stated that the decrease in MI can be caused by halting of G2 stage of the cell cycle, failure of removing free radicals or inhibition of DNA replication. MI reduction in fungicide treated-Group 4 should suggest that diniconazole is a mitodepressive compound. Also, it should be noted that the decrease of the mitotoic index with the increase in chromosomal abnormalities clearly demonstrates that the dinoconazole is cytotoxic. MI decline in *A. cepa* roots following diniconazole application was consistent with the other studies showing that triazoles are suppressive agents for mitotic division^38^.

**Table 3:** The effect of CME against diniconazole genotoxicity.

Damage	Group 1	Group 2	Group 3	Group 4	Group 5	Group 6
MN	0.56 ± 0.64^d^	0.42 ± 0.54^d^	0.24 ± 0.38^d^	35.70 ± 5.13^a^	27.80 ± 4.85^b^	19.50 ± 3.92^c^
MI	754.60 ± 25.94^a^	765.30 ± 26.82^a^	776.40 ± 27.66^a^	430.50 ± 19.48^d^	490.60 ± 21.64^c^	550.20 ± 22.85^b^
FRG	0.00 ± 0.00^d^	0.00 ± 0.00^d^	0.00 ± 0.00^d^	60.80 ± 7.52^a^	50.30 ± 6.84^b^	38.20 ± 5.65^c^
SC	0.36 ± 0.48^d^	0.26 ± 0.34^d^	0.12 ± 0.26^d^	40.40 ± 4.86^a^	32.50 ± 4.34^b^	24.90 ± 3.76^c^
UDC	0.00 ± 0.00^d^	0.00 ± 0.00^d^	0.00 ± 0.00^d^	30.40 ± 3.74^a^	22.60 ± 3.26^b^	15.80 ± 2.88^c^
VN	0.00 ± 0.00^d^	0.00 ± 0.00^d^	0.00 ± 0.00^d^	21.40 ± 2.36^a^	15.80 ± 1.96^b^	9.40 ± 1.18^c^

*Data are shown as mean ± SD (n = 10). Group 1: control, Group 2: 0.5 g/L CME, Group 3: 1.0 g/L CME, Group 4: 100 mg/L diniconazole, Group 5: 0.5 g/L CME + 100 mg/L diniconazole, Group 6: 1.0 g/L CME + 100 mg/L diniconazole. For MN and CAs 1,000 cells were counted in each group and 10,000 cells for MI. *MN* micronucleus, *MI* mitotic index, *FRG* fragments, *SC* sticky chromosome, *UDC* unequal distribution of chromatin, *B* bridge, *VN* vacuole nucleus. The averages shown with different letters ^(a–d)^ in the same line are statistically significant (p < 0.05).

Group 5 and Group 6 treated with a mixture of CME and diniconazole showed a gradual decrease in all CA types (Table 3). Dose-dependency was observed in the reduction of chromosomal damages and the enhancement of MI. CME was previously shown to be a protector against cytotoxicity induced by external chemicals^44^. Dinda et al.^45^ reported that *Cornus* fruits have effective pharmacological features such as antioxidant, anticancer, anti-inflammatory and antidiabetic merits due to high anthocyanin contents. Deng et al.^46^ associated the antigenotoxic power of *C. mas* fruits with their phytochemical compounds such as cornusid, loganin and sweroside. These fruits are also valuable with their glycosidic iridoids which possess antigenotoxic potential without any side effects^47,48^.

MDA content and the total activities of SOD and CAT enzymes were examined in order to evaluate the biochemical effects of diniconazole and CME on *A. cepa* roots (Table 4). An increase in the MDA content, a bioindicator of oxidative stress in the cells, refers to the damage to cellular membranes in connection with the peroxidation of polyunsaturated fatty acids^22^. Different doses of CME did not lead a significant MDA increase in Group 2 and Group 3 when compared to the control (Table 4). On the other hand, MDA content of diniconazole-applied Group 4 was nearly 5.4 folds that of the control group. Dinconiazole-related membrane deterioration has been previously demonstrated with animal experiments^49^. MDA levels decreased with increasing doses of CME applied as a mixture with diniconazole in Groups 5 and 6. MDA content of Group 6 approximately halved when compared to Group 4. The healing power of CME applied simultaneously with toxic compounds inducing lipid peroxidation is well documented^50^. Since *C. mas* is a rich source of natural antioxidants^51^, it was not surprising that it had protective effects against degradation of membrane phospholipids.

**Table 4:** Biochemical changes caused by diniconazole and CME.

Groups	SOD (U mg^−1^ FW)	CAT (OD_240 nm_min g^−1^ FW)	MDA (µmol g^−1^ FW)
Group 1	40.80 ± 5.27^d^	0.66 ± 0.06^d^	3.50 ± 1.48^d^
Group 2	39.60 ± 5.16^d^	0.64 ± 0.06^d^	3.60 ± 1.52^d^
Group 3	41.30 ± 5.32^d^	0.62 ± 0.05^d^	3.40 ± 1.36^d^
Group 4	88.70 ± 8.76^a^	1.26 ± 0.42^a^	18.90 ± 3.24^a^
Group 5	79.10 ± 7.63^b^	1.08 ± 0.24^b^	13.50 ± 2.85^b^
Group 6	63.50 ± 5.96^c^	0.88 ± 0.09^c^	9.70 ± 2.13^c^

*Data are shown as mean ± SD (n = 10). Group 1: control, Group 2: 0.5 g/L CME, Group 3: 1.0 g/L CME, Group 4: 100 mg/L diniconazole, Group 5: 0.5 g/L CME + 100 mg/L diniconazole, Group 6: 1.0 g/L CME + 100 mg/L diniconazole. *SOD* superoxide dismutase, *CAT* catalase, *MDA* malondialdehyde. The averages shown with different letters ^(a–d)^ in the same column are statistically significant (p < 0.05).

Increased activity of antioxidant enzymes is another bioindicator of oxidative stress. SOD enzyme acts as a major warrior in suppressing the ROS-mediated toxicity by catalyzing the conversion of superoxide anions to hydrogen peroxide and oxygen. Another endogenous antioxidant enzyme, CAT, contributes to the elimination of oxidative imbalance by converting hydrogen peroxide to water and oxygen^52^. CME application did not induce significant changes in the total activities of SOD and CAT in Groups 2 and 3 (Table 4). However, SOD and CAT activities in Group 4 were 2.17 and 1.91 times those of the control. Similar to our study, Mohamed et al.^53^ showed a remarkable increase in CAT activity of *Gossypium hirsutum* plants following the treatment of diniconazole. Although studies on the antioxidant enzyme activities of untargeted organisms exposed to diniconazole are very rare, there are many studies showing the induction of SOD and CAT activities by other triazoles^54,55^. CME caused significant decreases in SOD and CAT activities when applied with diniconazole (Table 4). Considering the decrease levels in enzyme activities of Group 5 and compared to Group 4, it can be said that the positive effect of CME on antioxidant enzyme activities depends on the dose of CME in the mixture. *C.mas* fruits have been emphasized as a priceless source of dietary antioxidants and anthocyanins those contribute to the therapeutic functions of these fruits to improve the antioxidant response and prevent cells from the detrimental effects of oxidative stress^56,57^. Anthocyanins, iridoids, phenolic acids, flavonoids, and tannins are the five important structural groups found in *C. mas* fruits^58^. Reduction of SOD and CAT activities in the last two groups may be interpreted as the role of CME in helping antioxidant enzymes through mentioned components against ROS-mediated stress caused by diniconazole.

**Figure 2:**
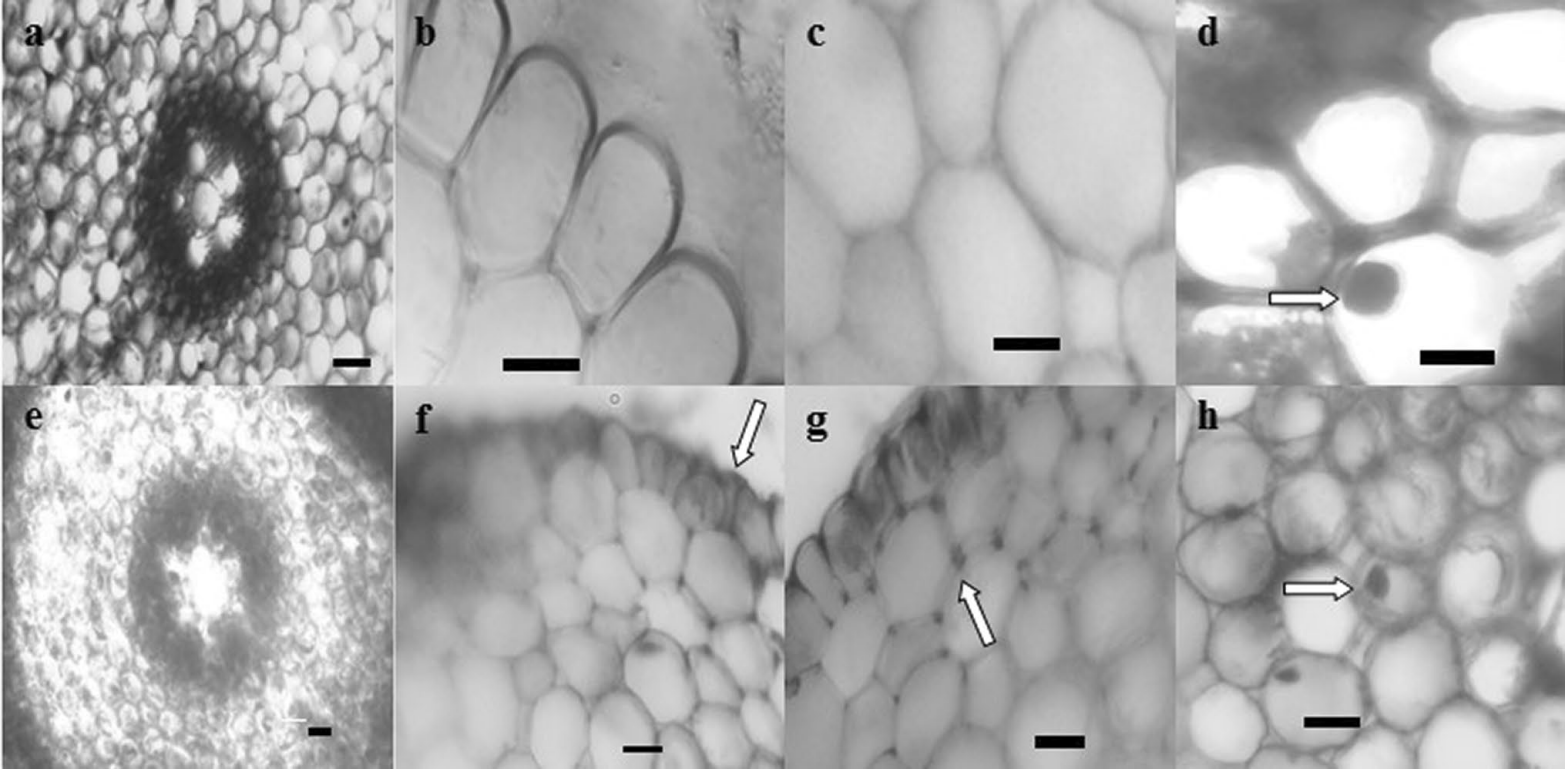
Meristematic cell damage. (**a**) Normal view of the transmission tissue, (**b**) normal appearance of epidermis cells, (**c**) normal view of cortex cells, (**d**) normal view of the cell nucleus, (**e**) indistinct transmission tissue, (**f**) epidermis cell deformation, (**g**) thickening of the cortex cell wall, (**h**) flattened cell nucleus) [Scale bar = 100 μm (**a**, **e**), Scale bar = 50 μm (**b**)–(**d**), (**f**)–(**h**)].

Effects of diniconazole and CME administrations on meristematic cells of *A. cepa* roots were checked microscopically. The appearance of the meristematic cells of the control group in cross-section was accepted as “normal” (Fig. 2a–d). In the meristematic cells of the CME treated groups (Groups 2 and 3), no damage was observed similar to the control group. On the other hand, damage levels observed in other groups were classified as “little”, “moderate” and “severe”. From this view of point, diniconazole caused a great damage to meristematic cells that the damage has been described as "severe" (Table 5). The damages observed following diniconazole application in *A. cepa* root meristematic cells were indistinct transmission tissue, epidermis cell deformation, thickening of the cortex cell wall and flattened cell nucleus (Fig. 2e–h). Epidermis deformation occurred probably due to squeezing of the cells forming outer layer of the roots in order to prevent the intake of diniconazole. Thickening of the cortex cell walls may be attributed to a strategy to reduce horizontal transport of the fungicide. In addition to its genotoxic effects within the nucleus, diniconazole caused the cell nucleus to show deformity. The structural integrity loss in transmission tissue may hinder the transfer of water and nutrients between the roots and the upper parts. This situation indicates that exposure to overdoses of dinconazole can be very risky for the vital activities of plants. Anatomical changes in meristematic cells may also be related to ROS, which attacks the structural components of cells and tissues and occurs due to exposure to diniconazole. Our results were in line with Demirtaş et al.^23^ who previously showed the diniconazole-mediated meristematic damages in *A. cepa* roots. In addition, Barnes et al.^59^ reported that triazoles including paclobutrazol and uniconazol led to disorganized anatomical structure in leaves and roots of *Zea mays* and *Glycine max*. Similarly, paclobutrazol induced distinct anatomical alterations in stem, leaf and roots of *Solanum tuberosum*^60^. 0.5 g/L CME lightened all malformations to “moderate” level in Group 5 and the intensity of the meristematic damages switched to “little” in Group 6 in which CME dose was increased to1.0 g/L. To the best of our knowledge, the restorative effect of CME in meristematic cells exposed to toxic materials has been reported for the first time in the literature. The healing role of CME in meristematic cells may be related to antioxidant wealth of *C. mas* fruits, which are highly effective in reducing potentially dangerous ROS for cells and tissues.

**Table 5:** Effects of diniconazole and CME applications on meristematic cells.

Damage	Indistinct transmission tissue	Epidermis cell deformation	Thickening of the cortex cell wall	Flattened cell nucleus
Group 1	−	−	−	−
Group 2	−	−	−	−
Group 3	−	−	−	−
Group 4	+++	+++	+++	++
Group 5	++	++	++	+
Group 6	+	+	+	+

*Group 1: control, Group 2: 0.5 g/L CME, Group 3: 1.0 g/L CME, Group 4: 100 mg/L diniconazole, Group 5: 0.5 g/L CME + 100 mg/L diniconazole, Group 6: 1.0 g/L CME + 100 mg/L diniconazole. (−): no damage, (+): little damage, (++): moderate, (+++): severe damage.

## Conclusions

Here, the toxicity of diniconazole fungicide as well as preventive efficiency of CME to alleviate dinoconazole-induced damages were investigated in *A. cepa* roots. Data obtained from the current study clearly demonstrated the detrimental potential of the fungicide in terms of physiological, cytogenetic and biochemical events as well as the fungicide-related damages in meristematic cells. MDA accumulation and reduction of the SOD and CAT activities as well as thickening of the cell wall following diniconazole treatment were reported for the first time in *A. cepa* in the present study. The destructive effects of diniconazole were substantially prevented by the addition of CME to the fungicide. The present work is the first report that revealed the therapeutic and protective efficacy of CME against diniconazole. The fact that the groups only administered CME showed similar results to those of the control revealed that the extract had no side effects. *A. cepa* has been shown to be a promising model for studies focusing on the toxic power of fungicides as well as the healing effects of medicinal plants. Dose–effect relationship between CME and its therapeutic capacity was stunning considering all parameters investigated. *C. mas* fruits should be moved to the upper steps in daily nutrition thanks to the antioxidants they contain. The protective functions of these laudable fruits should be investigated against other toxic substances.
